# Estimated number of deaths directly avoided because of COVID-19 vaccination among older adults in Colombia in 2021: an ecological, longitudinal observational study

**DOI:** 10.12688/f1000research.109331.3

**Published:** 2022-07-07

**Authors:** Maylen Liseth Rojas-Botero, Julián Alfredo Fernández-Niño, Leonardo Arregocés-Castillo, Fernando Ruiz-Gómez

**Affiliations:** 1 Ministerio de Salud y Protección Social, Bogota, Bogota, 110311, Colombia; 2 Universidad del Norte, Barranquilla, Colombia

**Keywords:** SARS-CoV-2, COVID-19, COVID-19 Vaccines, Aged, Mortality, Colombia.

## Abstract

**Background:** Colombia’s national COVID-19 vaccination plan began in February of 2021. It gave priority to older adults, who constituted 77.7% of deaths from this illness in the year 2020. The main goal of the plan is to decrease specific mortality and the number of serious COVID-19 cases, however, the number of deaths avoided by this strategy is unknown. The objective of this study was to estimate the number of avoided deaths in Colombia by fully vaccinating older adults against COVID-19, during the first year of the implementation of the national vaccination plan.

**Methods:** This study took on the design of an ecological, longitudinal study. Full vaccination coverage for older adults was calculated for each epidemiological week and age group from March to December 2021, based on which the number of avoided COVID-19 deaths was estimated. A sensitivity analysis was performed taking into account variations in the vaccines’ effectiveness by age group.

**Results:** In Colombia, over 5.3 million adults 60 years of age and older received full COVID-19 vaccinations between March and December 2021. During that same period, nearly 46,000 deaths of older adults from this cause were registered. We estimated that vaccination has avoided around 22,000 more older adults from dying from COVID-19 in Colombia, that is, 32.4% of expected deaths in 2021. According to the sensitivity analysis, the number of lives saved ranged from 19,597 to 36,507.

**Conclusions:** Colombia’s strategy to vaccinate older adults against COVID-19 has avoided mortality for this age group from being 48.0% higher than what was observed during the study period. Even more lives have been saved when taking into account the parameters that were defined and the omission of the contribution from partial vaccinations.

## Introduction

The COVID-19 pandemic has caused some of the worst social, economic and health crises in recent history, which poses unprecedented challenges for public health worldwide. ^1^ The number of people affected by the SARS-CoV-2 virus increased rapidly, with 297.4 million cases having been registered globally as of December 2021, and at least 5.4 million deaths. ^2^ In the case of Colombia, 5.1 million cases and roughly 130,000 deaths were reported during this same period. ^3^

While this illness can occur at any age, it has been found that older adults have an increased risk of negative outcomes from COVID-19, such as serious illness, hospitalization and death. ^4^^,^^5^ In fact, 77.7% of the deaths registered in Colombia during the year 2020 were adults 60 years of age and older. ^3^ Given the recognition of the greater load of serious morbidity and death, it has been recommended to prioritize older adults when implementing prevention measures. ^6^

Thanks to global efforts and academic, industry and government groups, successful vaccines have been developed at an unprecedented rate. Given these advances, current vaccinations, along with non-pharmacological measures, have become the best strategy for sustainably controlling the COVID-19 pandemic. ^7^

Colombia’s national COVID-19 vaccination plan was implemented on February 17, 2021. ^8^ Since it was first proposed, the Ministry of Health prioritized older adults, beginning with those 80 years of age and older, and making access to the vaccines progressively available based on higher risks of complication and death caused by COVID-19. ^8^

The first full vaccinations of older adults in Colombia were registered as of the second week of March, 2021 (epidemiological week 10) after the first vaccines to this age group were administered by the end of February, 2021. ^9^ In Colombia, the National Vaccination Plan had two great moments: the first focused on prioritizing people with the highest risk of complications and death in order to maximize the benefit of the vaccines, first guaranteeing access to people with the highest risk, and subsequently, this access was progressively opened to lower risk groups until total massification. In the case of older adults, the opening was given by five-year groups from older to younger during the first two (of five) stages. ^8^ Additionally, in the case of dispersed municipalities, it was also necessary to unify stages and complement them with active search strategies at home.

As part of the evaluation of the national vaccination plan, Esperanza cohort, a population-based-study estimated the effectiveness of the COVID-19 vaccines in older adults according to laboratory and age group. ^10^ Nevertheless, the number of deaths that were avoided among older adults in Colombia was still unknown at the end of the year.

Therefore, the purpose of this study was to estimate the number of deaths that were avoided by fully vaccinating the population of adults 60 years of age and older in Colombia in 2021, between epidemiological weeks 10 and 52.

## Methods

### Study design and population

An observational, ecological, population-based longitudinal study was performed. The unit of analysis corresponded to the country during the time between epidemiological weeks 10 and 52, in the year 2021. This analysis only considers the coverage of complete vaccination schedule (we excluded those partially vaccinated) among all older adults (aged 60 years and over) residing in Colombia in 2021. No other exclusion criteria were applied.

This analysis included information from 5340863 older adults registered as fully vaccinated in Colombia during 2021. Given the lags in reporting to information systems, the actual number of vaccinated would be higher.

### Information sources

This study used the weekly number of full COVID-19 vaccinations for adults ages 60 to 110 years old, according to age group. These data were managed by the PAIWEB information system of the Ministry of Health and Social Protection (MinSalud in Spanish).PAIWEB records the application of the doses since the beginning of the distribution of the vaccines against COVID-19 in Colombia. The study also used the number of weekly COVID-19 deaths according to simple ages, made available by the Integrated Social Protection Information System (SISPRO) and it is publicly available through the Colombian government's open data page. In addition, population projections for the year 2021 were used, available through DANE, ^11^ as was the vaccines’ effectiveness in preventing deaths by age group, as reported for Colombia. ^10^

### Data analysis

The results are presented with texts and figures. The analysis was performed with Excel ^®^ (Microsoft corporation, 2019) (RRID:SCR_016137) and STATA ^®^ 16.1 for Mac (StataCorp. 2019) (RRID:SCR_012763).

### Statistical analysis

First, the evolution of full vaccination coverage among older adults was presented by age groups: 60-69, 70-79 and 80 and older. Coverage was calculated using the population projection at the midpoint of the period as the denominator, for each age group. Full vaccination was defined as one dose of Ad26.COV2.S (Johnson & Johnson) or two doses of BNT162b2 (Pfizer), ChAdOx1 (AstraZeneca) nCoV-19, CoronaVac (Sinovac) or mRNA-1273 (Moderna). ^12^

The number of COVID-19 deaths that were avoided for fully vaccinated adults 60 years and older was then estimated for epidemiological weeks 10 through 52 of 2021 using the methodology proposed by Machado *et al.* ^13^ and previously used to estimate number of deaths directly averted in people 60 years and older as a result of COVID-19 vaccination in Europa. ^14^

The number of avoided deaths was estimated as follows:

$$D_{a}=\sum_{\mathrm{ij}}\left[D_{o_{\mathrm{ij}}}\times \frac{{\mathrm{FV}}_{i-2,j}\times {\mathrm{VE}}_{j}}{1-\left({\mathrm{FV}}_{i-2,j}\times {\mathrm{VE}}_{j}\right)}\right]$$

where $D_{a}$ corresponds to the avoided deaths, $D_{o_{\mathrm{ij}}}\;$ corresponds to the observed deaths during week i , age group j , ${\mathrm{FV}}_{i-2}$ corresponds to full vaccine coverage two weeks before week i for age group j , and ${\mathrm{VE}}_{j}$ corresponds to the vaccines’ effectiveness for preventing COVID-19 deaths for age group j .

As mentioned previously, the national vaccination plan was implemented on February 17, 2021. ^15^ Therefore, the first full vaccinations were observed beginning on March 7. ^9^ Thus, i ranges between epidemiological weeks 10 and 52 of the year 2021.

In addition, the time it takes for the vaccines to generate immune protection was considered, in this case, approximately 14 days. Thus, week i−2 corresponds to the lag time between vaccination coverage and the clinically relevant time for vaccine protection.

The effectiveness of the vaccines in preventing death was based on the Esperanza cohort study, ^10^ which reported 87.6% for adults ages 60 to 69 years old, 78.9% for those ages 70 to 79 years, and 61.2% for adults 80 years of age and older. These effectiveness rates are lower than those found for preventing death after hospitalization ^10^ and were selected in order to obtain conservative estimates of the number of avoided deaths.

After estimating the avoided deaths with the strategy, the number of expected deaths $D_{\exp}$ was calculated for an unvaccinated scenario, as follows ^13^:

$$D_{\exp}=D_{o}+D_{a}$$

This information was used to graph expected versus observed mortality rates per 100,000 using, as the denominator, the population projection as of the midpoint of the period for each age group. In addition, the preventable fraction was calculated as the proportion of the number of deaths observed with respect to expected deaths.

Following on from Machado’s *et al. study*, the number needed to be vaccinated (NNV) to avoid a death was estimated as ^13^:

$${\mathrm{NNV}}_{j}=\frac{1}{{\mathrm{VE}}_{j}\times \frac{D_{\mathrm{oj}}+D_{\mathrm{aj}}}{{\text{Population}}_{j}}}$$

*Sensitivity analysis*

The analysis was replicated from the Esperanza cohort’s report ^9^ of the lowest and highest effectiveness rates for preventing death without prior hospitalization, for each age group. The age groups were based on those defined originally in the Esperanza cohort. ^9^ The number of avoided deaths was calculated for an effectiveness of 82.5% and 95.0% for the 60–69-year-old group, an effectiveness of 70.7% and 95.7% for the 70–79-year-old group, and 59.1% and 83.4% for the 80 years and older age group. ^10^

### Ethical considerations

This investigation meets the scientific, technical, administrative, and ethical considerations stipulated by existing regulations for research with human beings in Colombia. In accordance with 1993 resolution 8430, this investigation is classified as no risk given its exclusive use of aggregated and secondary information sources. None of the study researchers accessed the databases with the original personal identifiers, and only the anonymized databases. All information handling standards were followed. Due to these characteristics, this study did not require review or approval by a research ethics committee.

The Ministry of Health and Social Protection is governed by national legislation on information management, habeas data laws, and institutional manuals of good practices. All information sources are directly managed by the Ministry, and the bases are anonymized, joined, and consolidated by an independent technician, through the generation of their own encrypted key code that allows the sources to be linked without using the original personal citizen identification. In this way it is not possible for researchers or external agents to recover the original identity numbers or personal data.

## Results

**Figure 1.**
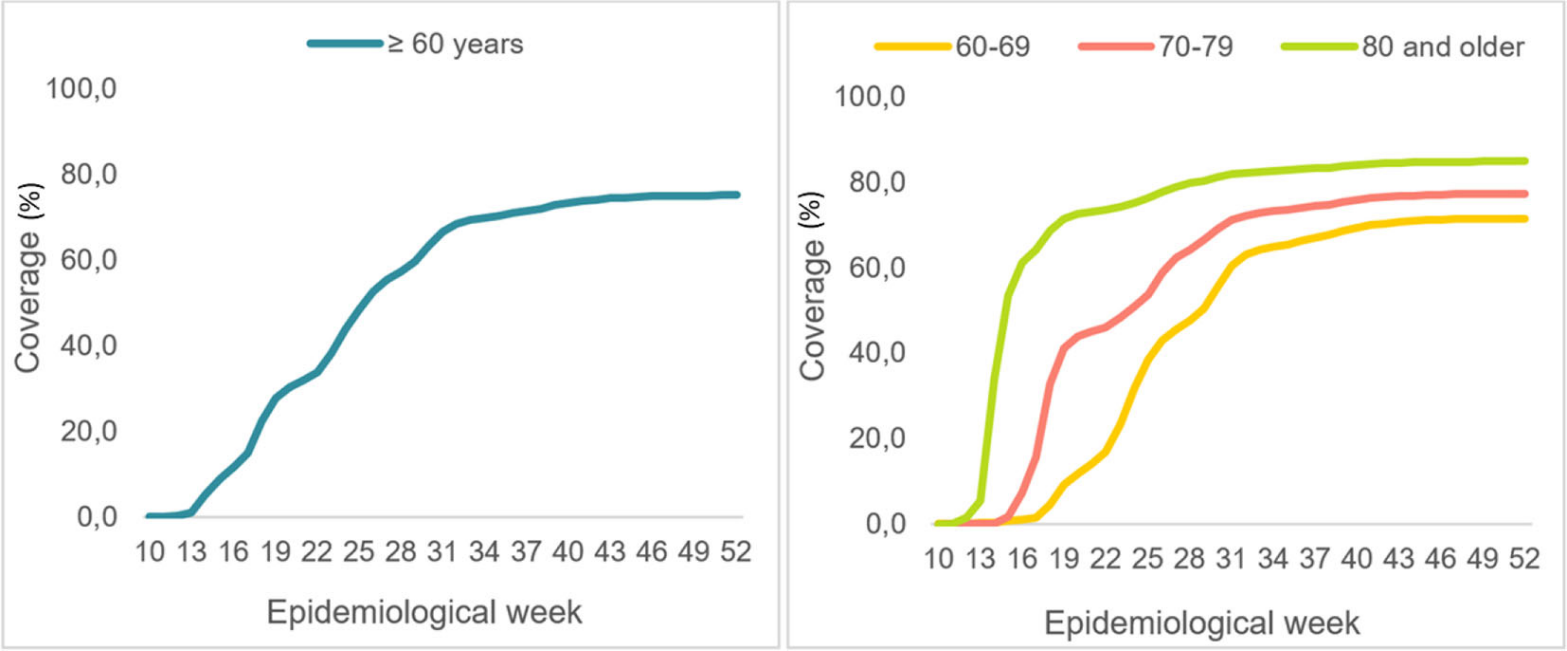
Evolution of full COVID-19 vaccination coverage for older adults. Colombia. Epidemiological weeks 10 to 52, of 2021.

Between March and December of 2021 (epidemiological weeks 10 through 52), over 5.3 million adults 60 years and older in Colombia received full COVID-19 vaccinations. As the vaccination strategy progressed ( Figure 1) 3,45,983 COVID-19 deaths were recorded for this population, for a specific mortality rate of 646.9 per 100,000 for that epidemiological period ^16^ ( Table 1).

**Table 1.** COVID-19 avoided deaths by being fully vaccinated for adults 60 years of age and older. Colombia. Epidemiological weeks 10 to 52 of 2021.

Age group	Effectiveness of full vaccinations	Vaccination coverage (%)	Deaths observed	Avoided deaths	Observed mortality rate per 100,000	Specific expected mortality per 100,000	Preventable fraction (%)
60-69	87.6	71.5	16,503	4,570	584.2	745.9	21.7
70-79	78.9	77.2	15,431	7,889	733.1	1,107.9	33.8
80+	61.2	84.8	14,049	9,619	1,338.3	2.254.7	40.6
**60 +**	--	--	45,983	22,078	646.9	957.5	32.4

As seen in Figure 1, the population 80 years and older was vaccinated more quickly, with over 70% full vaccination coverage for this age group by epidemiological week 19 (May 9-15, 2021). In contrast, adults 60 to 69 years old reached that level of coverage on epidemiological week 41 (October 10-16, 2021). ^16^

The weekly number of avoided deaths by full vaccination of adults 60 years and older was estimated. Using effectiveness indicators for each age group, it was estimated that 32.4% of the total expected deaths of adults 60 years of age and older was avoided by full vaccinations during the study period (a total of 22,078 lives saved), ranging from 21.7% of deaths avoided in the population 60 to 69 years old to 40.6% in those 80 years and older ( Table 1).

However, according to the sensitivity analysis, the total number of lives saved ranged from 19,597 to 36,507 when used the lowest and highest effectiveness rates for preventing death for each age group.

**Figure 2.**
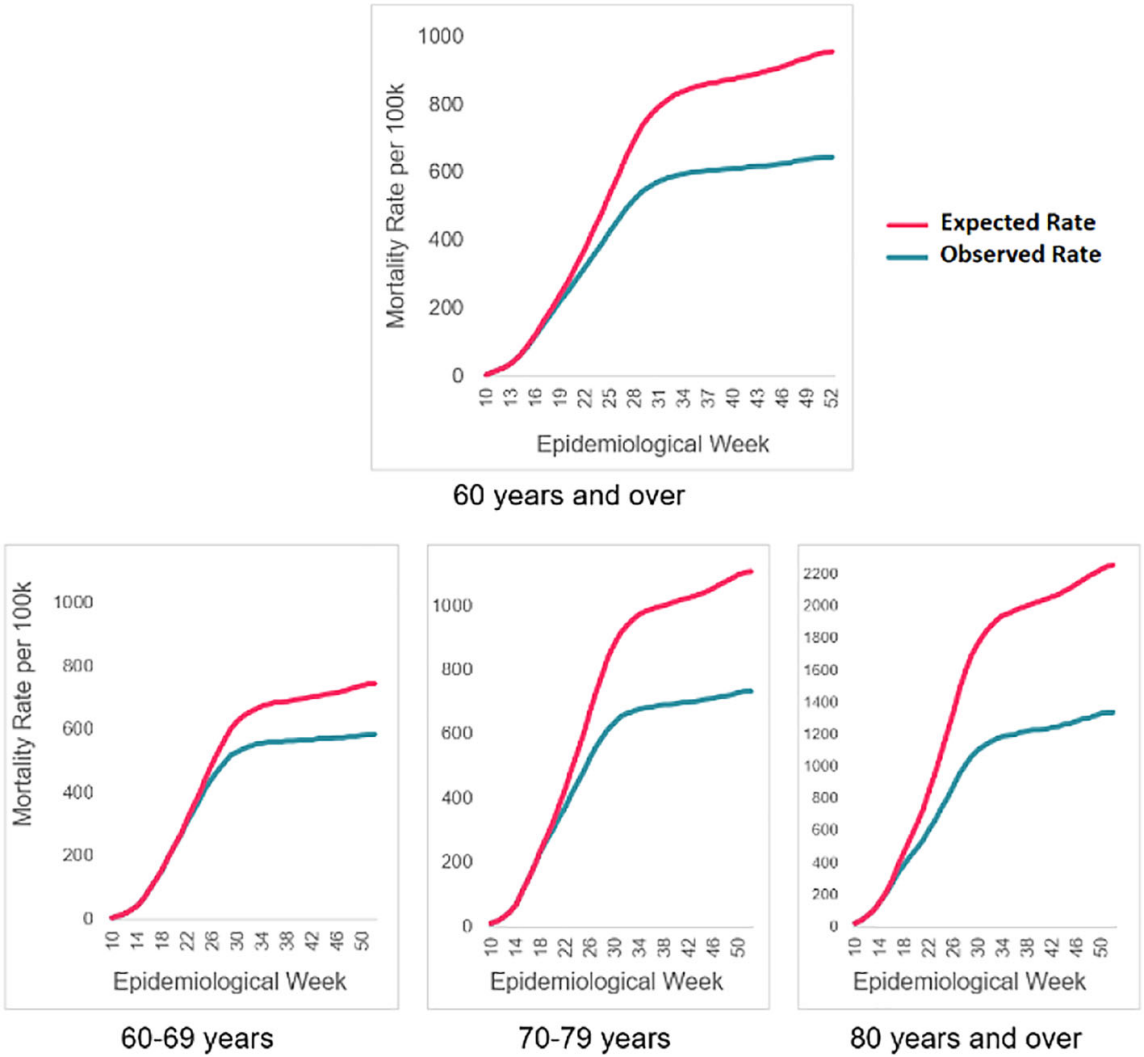
Accumulated COVID-19 mortality rates expected vs observed. Avoided deaths for adults 60 years of age and older as of vaccination. Colombia, 2021.

The largest preventable fraction was found among adults 80 years of age and older. In a scenario without COVID-19 vaccinations, the expected mortality rate would be 2,254.7 deaths from COVID-19 per 100,000 inhabitants for this age group during the observation period. Nevertheless, the observed rate was 40.6% lower, with 1,338.3 deaths per 100,000. As shown in Figure 2, the number of observed deaths is lower than the number of expected deaths as of epidemiological week 17.

Lastly, the number needed to be vaccinated to prevent a death was estimated at 166 for the 60- to 69-year-old group, 136 for those between 70 to 79 years old and 77 for adults 80 years of age and older.

## Discussion

The results of this preliminary analysis suggest that Colombia’s national COVID-19 vaccination plan has avoided at least 22,000 deaths of older adults between epidemiological weeks 10 and 52 in its first year of implementation.

Colombia designed a plan based on risk prioritization criteria, in which the first two stages included adults 60 years of age and older, beginning with those who were at least 80 years old—along with health personnel. ^8^ The strategy that was defined made it possible to attain a high vaccination coverage more quickly for this age group than for younger people. As a result of this decision, which was based on ethical and epidemiological principles, ^8^ a greater number of lives could be saved given that older adults constitute the group with the greatest mortality load. ^5^ Protecting them first optimized the benefits that immunization provides for specific mortality. In addition, this initial prioritization led the way for this group to be the first to receive the booster dose.

Of the 22,078 deaths that were prevented among adults 60 years of age and older, 43.6% were avoided among those who were 80 years old and over. Similar results were reported by Meslé *et al.* ^14^ who found that adults 80 years and older constituted 57.1% of the deaths that were avoided in 30 countries in Europe due to partial and full COVID-19 vaccinations. These findings affirm the importance of prioritizing older adults when implementing prevention measures.

In the same study, ^14^ the researchers found that between December 2020 and November 2021, COVID-19 vaccinations prevented 469,186 deaths of older adults (51.5% of expected deaths) in 33 countries in Europe, with preventable fractions ranging from 5.6% in Ukraine to as much as 92.9% in Iceland. These results are related with the speed with which the countries achieved high vaccination coverage for adults 60 years of age and older. Specifically, in Moldova, Romania and Ukraine, where full coverage was under 60.0%, the highest preventable fraction was 19.9%, whereas countries that quickly achieved full coverage for over 95.0% of their population (Iceland and England) prevented at least 85.8% of expected deaths. ^14^

While the number of deaths that were found to have been prevented in Europe is greater than that reported for Colombia, this comparison should be made cautiously. There are several reasons the studies may not be directly comparable for example, that study used effectiveness measures that were higher than what has been previously reported for older adults (60.0% for partial and 95.0% for full vaccinations). The estimates used in the present work are more precise since they take into account differences in the effectiveness of the vaccines by age group, although partial vaccinations are not included. If an estimate of 95.0% were to be used for protection from death with full vaccinations—such as in the study mentioned—then the number of avoided deaths in Colombia would double, reaching 46,214 lives saved.

This research offers a conservative measurement of the number of avoided deaths by vaccinating older adults in Colombia, given that it only included lives saved by full vaccinations and it used the lowest available effectiveness measures for each age group, which corresponded to the deaths without prior hospitalization that were reported in the Esperanza cohort. ^10^ In addition, the effectiveness of boosters was not considered, nor was a significant proportion of older adults who had hybrid immunity, whose protection would be greater. ^17^ Thus, the number of avoided deaths with the implementation of the national vaccination plan could be much higher than the estimates reported herein.

The limitations of this study include those that are inherent to the use of secondary information sources, such as the lag between registering events such as vaccinations and deaths. In addition, since this is an ecological analysis, the results could vary when considering individual and ecological variables with which counterfactual scenarios are projected. ^18^ In addition, in Colombia, the coverage with boosters only began at the end of November, so its effects on the prevention of deaths could not be estimated in this first analysis.

Additionally, this study only considered confirmed COVID-19 deaths, and certainly some COVID-19 deaths went undetected. Although, according to national guidelines, applying a diagnostic test for clinical suspicious deaths (including post-mortem test) is mandatory in Colombia, some people may not have had access to a diagnostic test before or after they died. Furthermore, for any cause of death, there will always be issues related to the final classification of the leading cause of death, as it also depends on access to medical care, clinical judgment, and ultimately the quality of death certificates.

In addition, it is important to distinguish “deaths with COVID-19” from “deaths from COVID-19”, and as matter of fact for some deaths it may be difficult to identify the actual causal chain that led to death even when there is a positive diagnostic test.

However, it is well known that actual deaths from COVID-19 are higher, given the deaths that go undetected. The use of suspicious deaths could increase the estimate of deaths prevented, but it could also overestimate the number of avoided deaths. For now, it can be mentioned that Colombia, unlike other countries, has a high convergence between the rate of confirmed mortality and the excess mortality rate, which is an indicator of the good quality of the mortality registry. ^19^

People from some areas and of some socioeconomic level may have greater barriers to access diagnostic tests before or after death, in addition, there are surely variations in the quality of death records throughout the country. These issues could lead to an underestimation of the number of deaths related to COVID-19, but also the estimated number of deaths directly averted. More studies are needed to adjust these estimates considering the variation in the quality of death certificates.

On the other hand, the prevention of deaths is certainly multifactorial, and non-pharmacological interventions (NPI) likely also played a role, especially during the early phases of the pandemic. The impact of these other measures (NPI) is not evaluated in this study, and to do so would require another and more complex approach, which is beyond our objectives.

Nevertheless, this approach enables comparisons with previous analyses of other countries, and constitutes one of the components in the comprehensive evaluation of the national vaccination plan, which has as one of its main objectives as the reduction of specific mortality. ^15^ Another strength of this work is the use of vaccine effectiveness measures estimated in real-life conditions for Colombia’s older adult population for each of the age groups, ^9^ thereby providing results that are closer to the specific situation of the country.

Future studies could evaluate the number of avoided deaths for the entire population or for special groups, such as children, across the different stages of implementing the national vaccination plan, which is continuing into the year 2022. The scientific and academic communities are encouraged to use other approaches to evaluate and compare the consistency of the conclusions. Lastly, this work serves as input for continuing to promote vaccinations as one of the key strategies for overcoming the COVID-19 pandemic, especially in the older adult population. ^20^

## Data availability

### Source data

All the original data used to build the final dataset of this study are of public access and can be downloaded from the official websites. The links to access them are shown below:

The dataset with all the COVID-19 confirmed cases and deaths in Colombia at an individual level is public available at the following link: https://www.datos.gov.co/widgets/gt2j-8ykr?mobile_redirect=true.

This database has individual and anonymous information on each confirmed case of COVID-19 in Colombia. For each case, the date of onset of symptoms, the date of report, sex, age, and final clinical status (dead or alive) are presented.

Board that allows consultation of vaccination coverage for COVID-19 by epidemiological week, municipality and age group in Colombia.

### Extended data

**Figshare:** Dataset of Study: Estimated number of deaths directly avoided because of COVID-19 vaccination among older adults in Colombia. https://doi.org/10.6084/m9.figshare.19122530 ^16^

This project contains the following extended data:

- Dataset Avoided Deaths (aggregated data used for final analysis)

The authors have permission to publish data from the databases used or if the data were under open licenses that allowed republication.

Data are available under the terms of the Creative Commons Attribution 4.0 International license (CC-BY 4.0).
