# Supplementary material for: Estimated number of deaths directly avoided because of COVID-19 vaccination among older adults in Colombia in 2021: an ecological, longitudinal observational study
Source: F1000Res. 2022 Jul 7;11:198. Originally published 2022 Feb 16. [Version 3] doi: 10.12688/f1000research.109331.3 (PMC9214267; doi:10.12688/f1000research.109331.3)
Supplement: Peer review history

# Peer review: Reviewer response for version 2

**DOI:** 10.5256/f1000research.134136.r140225

**Published:** 2022-06-21

**Authors:** Jenny Garcia

**Affiliations:** 1 French Institute for Demographic Studies (INED), Université Paris 1 Panthéon Sorbonne, Paris, France

The authors used a counterfactual analysis to estimate the impact of the COVID-19 vaccination strategy on avoiding deaths among older adults in Colombia. This is an important study because a) it quantifies the expected number of deaths without intervention and the estimated number of avoided death when considering the features of the vaccination campaign deployed in Colombia, and b) it contributes to the ongoing debate on the impact of the vaccination strategies deployed by the countries in the onset of their campaigns. The major strength of this study is the usage of the most up-to-date data on vaccination coverage by age groups related to observed vaccine effectiveness in the same population. The authors applied rather simple methods to develop an easily replicable study. Both data and methods are well-referenced and available. While the results are compelling, some comments rose during the review of this research:

1. According to the authors, the vaccination campaign in Colombia started at the end of February 2021. The share of fully vaccinated people increased rapidly, and around 60% of the population aged 80+ was fully vaccinated in the following five weeks (week 15 starting on April 12, 2021). According to ‘our world in data’, Colombia’s percentage of fully vaccinated people was 2.32% at the end of the epidemiological week 15 ( https://ourworldindata.org/covid-vaccinations). This pace of vaccination in the population aged 80+ outstrips the record of forerunners countries such as the USA ( https://www.cdc.gov/coronavirus/2019-ncov/vaccines/distributing/about-vaccine-data.html,) Belgium ( https://covid19-country-overviews.ecdc.europa.eu/countries/Belgium.html#vaccine-uptake), and Chile, where a similar share of complete vaccination in the population aged 80+ took several months or occurred when a larger percentage of people at all ages were fully vaccinated. The difference may come from the type of vaccines used by the countries, the difference in the vaccination strategies or the smaller size of the population aged 80+. According to PAHO figures ( https://ais.paho.org/imm/IM_DosisAdmin-Vacunacion.asp), the share of J&J vaccines used in Colombia was 9 out of 56 million, which means that most of the vaccines in use required two doses. I advise the authors to revise the calculation of the share of fully vaccinated people summarized in figure 1 adding the distribution of the fully vaccinated population by type of vaccine if one-dose or two-dose vaccines, as the scheme of complete vaccination by age group. A brief discussion on the vaccination strategies applied in Colombia is also advisable in this case.
2. Authors may calculate more precisely the number of avoided and expected deaths by including the observed vaccine effectiveness and the in-use share by type of vaccine. They have already estimated the vaccine effectiveness by type of vaccine in previous research (paper cited in the reference Arregoces Castillo et al. 2021 ^1^). I enquire the authors, why these estimations were not included if as shown variations were found?
3. Lastly, I suggest the authors revisiting their aim at using the indicator “number of fully vaccinated people needed to prevent a death”. As currently stated, a larger number of fully vaccinated people are needed to prevent the death of a person aged 60 to 69 than those who need to prevent the death of a person aged 80 and over. This result seems counterintuitive, especially considering that the raw counts and rates are higher, and the vaccine effectiveness is the lowest for the 80+ age group. In this sense, results are merely informative of the composition effect of the population, in which population size decreases among age groups. Thus, more detailed interpretations of these findings may be needed. In addition, when revising the original paper from which the applied method was taken (Machado et al. 2019 ^2^). Machado et al. targeted an international comparison of the number of observed and avoided medically attended influenza-confirmed cases rather than age-differentials in mortality. In this matter, I advise them to add also some discussion about the consequence of using death counts instead of cases for calculating this indicator in the discussion

Is the work clearly and accurately presented and does it cite the current literature?

Yes

If applicable, is the statistical analysis and its interpretation appropriate?

Yes

Are all the source data underlying the results available to ensure full reproducibility?

Yes

Is the study design appropriate and is the work technically sound?

Yes

Are the conclusions drawn adequately supported by the results?

Yes

Are sufficient details of methods and analysis provided to allow replication by others?

Yes

Reviewer Expertise:

Demography, Mortality, causes of death analysis using imperfect statistics, Latin America.

I confirm that I have read this submission and believe that I have an appropriate level of expertise to confirm that it is of an acceptable scientific standard.

## References

[1] : Effectiveness of COVID-19 Vaccines in Older Adults in Colombia: First Report of the Esperanza Cohort. A Matched-Pair, National Study. *SSRN Electronic Journal* .2021; 10.2139/ssrn.3978556 10.2139/ssrn.3978556

[2] : Impact of influenza vaccination programmes among the elderly population on primary care, Portugal, Spain and the Netherlands: 2015/16 to 2017/18 influenza seasons. *Euro Surveill* .2019;24(45) : 10.2807/1560-7917.ES.2019.24.45.1900268 10.2807/1560-7917.ES.2019.24.45.1900268 31718740PMC6852314

# Response

**Published:** 2022-07-05

**Authors:** Julián Alfredo Fernández-Niño

**Affiliations:** Universidad del Norte, Colombia

We thank the reviewer for her valuable comments. Here we find our responses:

**Comment**: 1. According to the authors, the vaccination campaign in Colombia started at the end of February 2021. The share of fully vaccinated people increased rapidly, and around 60% of the population aged 80+ was fully vaccinated in the following five weeks (week 15 starting on April 12, 2021). According to ‘our world in data’, Colombia’s percentage of fully vaccinated people was 2.32% at the end of the epidemiological week 15 (https://ourworldindata.org/covid-vaccinations). This pace of vaccination in the population aged 80+ outstrips the record of forerunners countries such as the USA (https://www.cdc.gov/coronavirus/2019-ncov/vaccines/distributing/about-vaccine-data.html,) Belgium (https://covid19-country-overviews.ecdc.europa.eu/countries/Belgium.html#vaccine-uptake), and Chile, where a similar share of complete vaccination in the population aged 80+ took several months or occurred when a larger percentage of people at all ages were fully vaccinated.

 The difference may come from the type of vaccines used by the countries, the difference in the vaccination strategies or the smaller size of the population aged 80+. According to PAHO figures (https://ais.paho.org/imm/IM_DosisAdmin-Vacunacion.asp), the share of J&J vaccines used in Colombia was 9 out of 56 million, which means that most of the vaccines in use required two doses. I advise the authors to revise the calculation of the share of fully vaccinated people summarized in figure 1 adding the distribution of the fully vaccinated population by type of vaccine if one-dose or two-dose vaccines, as the scheme of complete vaccination by age group. A brief discussion on the vaccination strategies applied in Colombia is also advisable in this case.

**Response:** Unfortunately, considering the reporting lag in *PAIWEB* is not possible to have updated coverages for each specific vaccine in Colombia at this point. These coverages will be available officially soon, and then we could update this same exercise.   

 Following the peer´s suggestion, we now included a brief discussion on the vaccination strategies in Colombia in the manuscript:

 “In Colombia, the National Vaccination Plan had two great moments: the first focused on prioritizing people with the highest risk of complications and death in order to maximize the benefit of the vaccines, first guaranteeing access to people with the highest risk, and subsequently, this access was progressively opened to lower risk groups until total massification. In the case of older adults, the opening occurred for five years from older to younger during the first two stages. Additionally, in the case of dispersed municipalities, it was also necessary to unify stages and complement them with active search strategies at home”

**Comment**: 2. Authors may calculate more precisely the number of avoided and expected deaths by including the observed vaccine effectiveness and the in-use share by type of vaccine. They have already estimated the vaccine effectiveness by type of vaccine in previous research (paper cited in the reference Arregoces Castillo et al. 20211). I enquire the authors, why these estimations were not included if as shown variations were found.

**Response:** The cited article was published by us. The reason for using the effectiveness estimate for all vaccines is that at that time and still now the specific consolidated coverage for each vaccine was and is not still publicly available. Therefore, we use the coverage given by people fully vaccinated as in other previous study.

 However, in the sensitivity analysis, the estimation exercise was performed by changing the coverage in a range between the lowest and the highest coverage estimated by age group.

 This exercise should be repeated with better consolidated information when it becomes available.

**Comment**: 3. Lastly, I suggest the authors revisiting their aim at using the indicator “number of fully vaccinated people needed to prevent a death”. As currently stated, a larger number of fully vaccinated people are needed to prevent the death of a person aged 60 to 69 than those who need to prevent the death of a person aged 80 and over. This result seems counterintuitive, especially considering that the raw counts and rates are higher, and the vaccine effectiveness is the lowest for the 80+ age group. In this sense, results are merely informative of the composition effect of the population, in which population size decreases among age groups. Thus, more detailed interpretations of these findings may be needed. In addition, when revising the original paper from which the applied method was taken (Machado et al. 2019 2). Machado et al. targeted an international comparison of the number of observed and avoided medically attended influenza-confirmed cases rather than age-differentials in mortality. In this matter, I advise them to add also some discussion about the consequence of using death counts instead of cases for calculating this indicator in the discussion.

**Response:** The reason that the NNT is lower with increasing age is mainly due to the increased risk of death with increasing age. Given an increased risk of complications and death in older adults with increasing age, the number of people needed to vaccinate is smaller. Similarly, if the risk is lower, more people need to be vaccinated, so the NNT decreases with age.

 Regarding the use of confirmed deaths, Finally, we included a comment in the discussion about the limitation of using confirmed deaths.

# Peer review: Reviewer response for version 2

**DOI:** 10.5256/f1000research.134136.r138429

**Published:** 2022-06-01

**Authors:** B. Piedad Urdinola

**Affiliations:** 1 School of Economics, Universidad Nacional de Colombia-Bogotá, Bogotá, Colombia

I have no further comments. Approved for indexing from my side.

Is the work clearly and accurately presented and does it cite the current literature?

Yes

If applicable, is the statistical analysis and its interpretation appropriate?

Yes

Are all the source data underlying the results available to ensure full reproducibility?

Partly

Is the study design appropriate and is the work technically sound?

Yes

Are the conclusions drawn adequately supported by the results?

Yes

Are sufficient details of methods and analysis provided to allow replication by others?

Partly

Reviewer Expertise:

NA

I confirm that I have read this submission and believe that I have an appropriate level of expertise to confirm that it is of an acceptable scientific standard.

# Peer review: Reviewer response for version 1

**DOI:** 10.5256/f1000research.120820.r128107

**Published:** 2022-05-11

**Authors:** B. Piedad Urdinola

**Affiliations:** 1 School of Economics, Universidad Nacional de Colombia-Bogotá, Bogotá, Colombia

The research question is a very important one that will allow us to understand the effects of vaccination on mortality for the most affected age group by the disease in Colombia and the proposed methods lead to a conservative estimation, however it can be further refined by adding the following comments:

1. The methods the authors proposed follow closely Machado et al.’s, that article explicitly exposes that they are accounting for the number of medically attended influenza-confirmed cases (MAICC). I believe this is also the case for the article at hand, accounting for medically attended cases, however it is not explicit in the document, and it makes an important difference to mention it. Because a part of the Colombian population inhabits low density municipalities with little or hard to reach health facilities where could be properly attended by a medical doctor. Besides other reasons why people pass unattended even with access to medical assistance, such as congestion due to the pandemic, postponement to visit MDs due to fear of contagion and the like.
2. On the same note, please make explicit if the paper is accounted deaths are all confirmed deaths or if they also include suspicious cases as well.
3. Finally, there could also be confounding with other government policies or self-imposed habits that reduce the mortality in the group under study. Particularly in Colombia, lockdowns were prolonged for elderly, and many may have changed their behavior even after the restrictions were removed. Also, the national protests occurred during the period under analysis and made very hard to move across some towns and even within certain towns such as Cali and Bogota, particularly for elderly who mostly were not involved in them. Authors could be inspired by the following work: Arbel, R., Moore, C. M., Sergienko, R., & Pliskin, J. (2022). How many lives do COVID vaccines save? Evidence from Israel. American journal of infection control, 50(3), 258-261. ^1^

Is the work clearly and accurately presented and does it cite the current literature?

Yes

If applicable, is the statistical analysis and its interpretation appropriate?

Yes

Are all the source data underlying the results available to ensure full reproducibility?

Partly

Is the study design appropriate and is the work technically sound?

Yes

Are the conclusions drawn adequately supported by the results?

Yes

Are sufficient details of methods and analysis provided to allow replication by others?

Partly

Reviewer Expertise:

NA

I confirm that I have read this submission and believe that I have an appropriate level of expertise to confirm that it is of an acceptable scientific standard, however I have significant reservations, as outlined above.

## References

[1] : How many lives do COVID vaccines save? Evidence from Israel. *Am J Infect Control* .50(3) : 10.1016/j.ajic.2021.12.019 258-261 10.1016/j.ajic.2021.12.019 34971714PMC8714257

# Response

**Published:** 2022-05-16

**Authors:** Julián Alfredo Fernández-Niño

**Affiliations:** Universidad del Norte, Colombia

We thank Dr. Urdinola for reviewing our manuscript. We present our responses below each comment as well as a new version of our manuscript (Version #2).

- The methods the authors proposed follow closely Machado et al.’s, that article explicitly exposes that they are accounting for the number of medically attended influenza-confirmed cases (MAICC). I believe this is also the case for the article at hand, accounting for medically attended cases, however it is not explicit in the document, and it makes an important difference to mention it. Because a part of the Colombian population inhabits low density municipalities with little or hard to reach health facilities where could be properly attended by a medical doctor. Besides other reasons why people pass unattended even with access to medical assistance, such as congestion due to the pandemic, postponement to visit MDs due to fear of contagion and the like.

**Response:** *Our analysis only included **confirmed **COVID-19 deaths. These deaths included some that were surely medically treated (the majority) and some that were not.*

*We use confirmed deaths obtained from the official national registry called RAUF-ND. Confirmed deaths includes deaths certified by a medical doctor regardless of whether the person was able to receive medical care before they died. COVID-19 death was defined as a death resulting from clinically compatible illness in a confirmed case of COVID-19, unless there was a clear alternative cause of death that could not be related to COVID-19. In this sense, Colombia uses the definitions recommended by the WHO for surveillance of COVID-19 (Pan American Health Organization Case definitions for COVID-19 surveillance. https://www.paho.org/en/case-definitions-covid-19-surveillance-16-december-2020).*

*Certainly, we know that some COVID-19 deaths went undetected as some people might not have had access to a diagnostic test before or after they died. (Although, according to national guidelines, applying a diagnostic test for clinical suspicious deaths (including post-mortem test) is mandatory in Colombia). Furthermore, for any cause of death, there will always be issues related to the final classification of the leading cause of death or even the previous clinical diagnosis, as it also depends on access to medical care, clinical judgment, and ultimately the quality of death certificates.*

*In addition, it is important to distinguish “deaths with COVID-19” from “deaths from COVID-19”, and as matter of fact for some deaths it may be difficult to identify the actual causal chain that led to death even when there is a positive diagnostic test.*

*We agree with the reviewer that this could be a big limitation of the study.*

*To address this, we need some adjustments that are not possible now considering the COVID death reclassification is a process that is still in progress. Colombia, like other countries in the world, is still advancing the process of reclassifying COVID-19 deaths by carrying out clinical algorithms, verbal autopsies, and the search for unidentified diagnostic tests. This is a process that takes months and ends in a process of statistical amendment. Therefore, we are thinking of repeating this same analysis when final classification is available.*

*We added this as a limitation in our discussion.*

  

- On the same note, please make explicit if the paper is accounted deaths are all confirmed deaths or if they also include suspicious cases as well.

***Response:** Only confirmed deaths from COVID-19 were considered in our analysis as in previous studies. Suspicious deaths include deaths where the cause cannot be confirmed to be associated with a SARS-Cov-2 infection, certainly this may include a proportion of real deaths from COVID-19, but also probably includes an important share of syndromatically similar deaths from other causes such as Influenza.*

*The use of suspicious deaths would imply making some risky assumptions about the proportion of suspicious deaths that would be COVID-19 deaths, which varies at the territorial level. This may be a later analysis as soon as we have the final reclassification of deaths in Colombia.*

*Now, that is mentioned in the discussion of the manuscript.*

  

- Finally, there could also be confounding with other government policies or self-imposed habits that reduce the mortality in the group under study. Particularly in Colombia, lockdowns were prolonged for elderly, and many may have changed their behavior even after the restrictions were removed. Also, the national protests occurred during the period under analysis and made very hard to move across some towns and even within certain towns such as Cali and Bogota, particularly for elderly who mostly were not involved in them. Authors could be inspired by the following work: Arbel, R., Moore, C. M., Sergienko, R., & Pliskin, J. (2022). How many lives do COVID vaccines save? Evidence from Israel. American journal of infection control, 50(3), 258-261.1

**Response**: *The estimate of avoided deaths made in this study is a conservative measure for reasons explained in the manuscript. However, the prevention of deaths is certainly multifactorial, and non-pharmacological interventions (NPI) likely also played a role, especially during the early phases of the pandemic as we showed before: https://www.ncbi.nlm.nih.gov/pmc/articles/PMC8289626/*

*In Colombia, the differential measures for older adults lasted only a few weeks after being suspended by the Colombian Constitutional Court in June of 2020 before of the beginning of the National Vaccination Plan in Colombia during 2021.   ( https://www.elespectador.com/salud/corte-constitucional-amparo-los-derechos-de-participantes-de-la-revolucion-de-las-canas/)*

*The impact of these other measures (NPI) is not evaluated in this study, and to do so would require another and more complex approach, which is beyond our objectives. However, we can affirm that the NPI (as national lockdowns) affected the entire population, although certainly in real life, some social groups could have different responses, for example to isolation whose adherence depends on employment or housing conditions (as well as other social determinants), however these factors are likely more important for the risk of infection than for the risk of death. Certainly, this study is a rough approximation from an ecological level, and it is not intended to evaluate the effect of other measures, nor their interaction with other factors.*

*Regarding the impact of the protests, it is certainly something very difficult to evaluate. We tried to explore this impact in one previous study:*

*https://papers.ssrn.com/sol3/papers.cfm?abstract_id=3899602*

*We added a comment in our discussion.*
